# Efficacy of robust optimization plan with partial‐arc VMAT for photon volumetric‐modulated arc therapy: A phantom study

**DOI:** 10.1002/acm2.12131

**Published:** 2017-07-10

**Authors:** Hideharu Miura, Shuichi Ozawa, Yasushi Nagata

**Affiliations:** ^1^ Hiroshima High‐Precision Radiotherapy Cancer Center Higashiku‐ku Hiroshima Japan; ^2^ Department of Radiation Oncology Institute of Biomedical & Health Science Hiroshima University Higashiku‐ku Hiroshima Japan

**Keywords:** arc angle, robust treatment planning, target position, volumetric modulated arc therapy, worst‐case scenario

## Abstract

This study investigated position dependence in planning target volume (PTV)‐based and robust optimization plans using full‐arc and partial‐arc volumetric modulated arc therapy (VMAT). The gantry angles at the periphery, intermediate, and center CTV positions were 181°–180° (full‐arc VMAT) and 181°–360° (partial‐arc VMAT). A PTV‐based optimization plan was defined by 5 mm margin expansion of the CTV to a PTV volume, on which the dose constraints were applied. The robust optimization plan consisted of a directly optimized dose to the CTV under a maximum‐uncertainties setup of 5 mm. The prescription dose was normalized to the CTV D_99%_ (the minimum relative dose that covers 99% of the volume of the CTV) as an original plan. The isocenter was rigidly shifted at 1 mm intervals in the anterior‐posterior (A‐P), superior‐inferior (S‐I), and right‐left (R‐L) directions from the original position to the maximum‐uncertainties setup of 5 mm in the original plan, yielding recalculated dose distributions. It was found that for the intermediate and center positions, the uncertainties in the D_99%_ doses to the CTV for all directions did not significantly differ when comparing the PTV‐based and robust optimization plans (*P* > 0.05). For the periphery position, uncertainties in the D_99%_ doses to the CTV in the R‐L direction for the robust optimization plan were found to be lower than those in the PTV‐based optimization plan (*P* < 0.05). Our study demonstrated that a robust optimization plan's efficacy using partial‐arc VMAT depends on the periphery CTV position.

## 1 INTRODUCTION

Intensity‐modulated radiation therapy (IMRT) and volumetric‐modulated arc therapy (VMAT) have great potential to deliver highly conformal tumor doses to targets while minimizing doses to any nearby organ at risk (OAR).^1^, ^2^, ^3^, ^4^ VMAT significantly improves delivery efficiency while maintaining a treatment‐plan quality similar to that of IMRT.^2^ Comparisons of the dosimetric and delivery efficiency between 360° single‐arc and 180°–200° partial‐arc VMAT have been made for ipsilateral cancer; these studies reported that partial‐arc VMAT significantly reduced the mean dose to the contralateral OAR with decreased delivery time.^3^, ^4^

Intensity Modulated Proton Therapy (IMPT) dose distributions are sensitive to both high stopping‐power dependency and steep beam‐dose gradients. The Bragg peak positions are highly affected by the densities and materials of the volume traversed by the incident protons. In proton therapy, setup errors, density errors, and organ motion can lead to differences in dose distributions when comparing the planned and delivered doses. Several authors have previously reported that stochastic programming and robust optimization in IMPT have minimized this problem.^5^, ^6^, ^7^, ^8^, ^9^, ^10^, ^11^, ^12^ Presently, RayStation (RaySearch Medical Laboratories AB, Stockholm, Sweden) offers one of the robust optimization methods used to address these demands. RayStation's robust optimization has been applied mainly to treatment planning for IMPT.

There are limited reports of robust optimization plans for photon treatment planning systems (TPSs).^13^, ^14^, ^15^ Chan, et al. demonstrated the potential of using a robust optimization methodology in IMRT treatment planning to improve the sparing of healthy tissue while maintaining tumor coverage in the intrafraction breathing motion uncertainty. Byrne, et al. used Raystation robust optimization for ensuring coverage of the breast clinical target volume (CTV) with setup variations and was found to be comparable to other established planning methods. The purpose of this study is to investigate the possibility of VMAT robust optimization in the homogeneity region. We investigated whether the planning target volume (PTV)‐based and robust optimizations show tumor position dependence using full‐arc and partial‐arc VMAT.

## 2 MATERIALS AND METHODS

**Figure 1:**
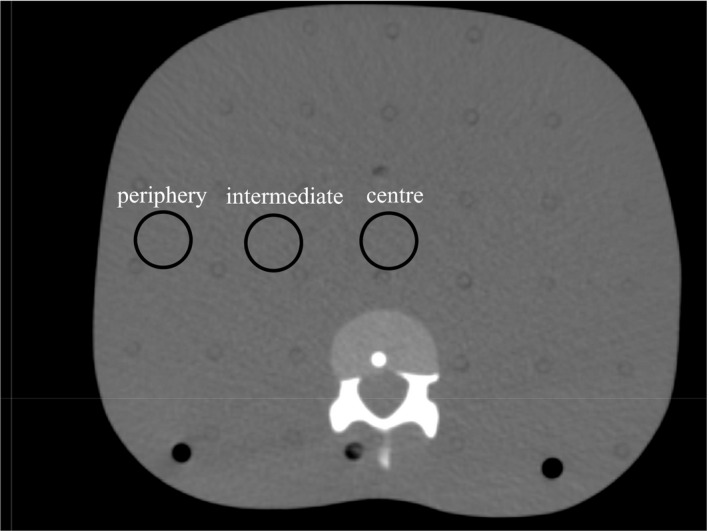
Locations of the three targets evaluated in this study. The target positions were defined as the periphery, the intermediate and the center positions, respectively. The intermediate and periphery positions are 4 cm and 8 cm offset locations from center position, respectively.

Anthropomorphic phantom was scanned with a computed tomography (CT) (Optima CT 580W; GE Healthcare, Milwaukee, WI, USA). Technique factors included a tube potential of 120 kV, a gantry rotation time of 0.5 s, a slice thickness of 1.25 mm and a tube current of 350 mA. A RayStation ver. 4.5 TPS was used for this study. Three sphere CTVs with diameters of 2 cm, that is, the periphery, intermediate position, and center of body (Fig. 1), which simulated of stereotactic body radiotherapy (SBRT), were investigated using an anthropomorphic phantom. The intermediate and periphery positions are 4 cm and 8 cm offset locations from center position, respectively. Photon beams with energies of 10 MV were used for all plans. A Varian TrueBeam STx (Varian Medical Systems, Palo Alto, CA, USA) linear accelerator was used for plan designs. The gantry angles for the plans at the periphery, intermediate, and center positions were set at 181°–360° (partial‐arc VMAT) and 181°–180° (full‐arc VMAT) clockwise arcs. The collimator angle was fixed at 10°. A PTV‐based optimization plan was defined by 5 mm margin expansion of the CTV to a PTV volume, on which the dose constraints were applied. The robust optimization plan entailed administering a directly optimized dose to the CTV under a maximum‐uncertainties setup of 5 mm. A PTV was not necessary for the robust optimization plan, but was used for an evaluative region of interest (ROI). We compared the PTV‐based optimization plan and the robust optimization plan to investigate the position dependence between full‐arc and partial‐arc VMAT. All the plans were created with a single fraction and a prescription dose of 1000 cGy.

The RayStation system offers minimax optimization, in which the optimization functions selected to be robust are considered under the worst‐case scenario.^8^ The interfractional patient‐setup uncertainties are considered to be random; they are incorporated by shifting the isocenter of the patient in the anterior‐posterior (A‐P), superior‐inferior (S‐I), and right‐left (R‐L) directions by the same margin as those used for defining the PTV. This yields six dose distributions, delineated by

$$\min_{x\in X}\max_{s\in S}\sum_{i=1}^{n}W_{i}f_{i}\left(d\left(x;\;s\right)\right)$$

where *W* is the weight, *f* is the function, *X* is the set of feasible variables, *d(x;s)* is the dose distribution as a function of the variables *x* and the scenario *s*, and *n* is the number of the scenario. Optimizing with robust objectives under several scenarios is an iterative process, and has the potential to require more iterations to get an equivalent objective function value.

The conventional TPSs for PTV‐based optimization are optimized to a PTV. For the robust optimization plan, the optimization goal for the CTV is directly optimized using maximum, minimum, and uniform dose constraints instead of a dose constraint for a PTV. The RayStation system offers both physically based (minimum dose, maximum dose, minimum dose volume histogram (DVH), maximum DVH, uniform dose, and uniformity) and biologically based (target equivalent uniform dose (EUD), minimum EUD, and maximum EUD) cost functions. The VMAT treatment plan was calculated using the collapsed cone convolution superposition (CCCS)‐based algorithm. The TPS dose grid size was 2.5 × 2.5 × 2.5 mm^3^. Byrne, et al. verified that RayStation robust optimization has an accurately model on the surface and at shallow depths using scintillator and film measurements.^15^

For quantitative comparisons based on the DVH, the dose distribution for each plan was normalized to that of a CTV D_99%_ (the minimum relative dose that covers 99% of the volume of the CTV) as an original plan. For the PTV, a homogeneity index (HI) was calculated using the following formula:^16^

$$\mathrm{HI}=\frac{D_{2\%}-D_{98\%}}{D_{50\%}},$$

where D_2%_, D_98%_, and D_50%_ are doses that covered 2%, 98%, and 50% of the PTV, respectively. The following method was used to investigate the variation in dose indices caused by setup errors. For setup uncertainties, the isocenter of the patient was rigidly shifted in the A‐P, S‐I, and R‐L directions, yielding six dose distributions. The isocenter was rigidly shifted from the original position to the maximum‐uncertainties setup of 5 mm at 1 mm intervals in the original plan. D_99%_ was used to evaluate the plan's quality, where the relative dose was the ratio of the received dose to the prescribed dose. The obtained data were analyzed using the analysis of variance (ANOVA) method, with the statistical significance set at *P* < 0.05.

## 3 RESULTS

In the original plan, Table 1 compares the HI to the PTV for PTV‐based and robust optimization plans using the full‐arc and partial‐arc VMAT plans, respectively. The values of the HI for the PTV‐based and robust optimization plans on the intermediate and center positions were almost the same using the full‐arc and partial‐arc VMAT plans, respectively. The HI values for the PTV‐based and robust optimization plans on the periphery position were, respectively, 0.015 and 0.040 (full‐arc) compared to 0.033 and 0.098 (partial‐arc). At the periphery position, the dose distribution of the PTV was more homogeneous for the PTV‐based optimization plan than for the robust optimization plan.

**Table 1:** Comparison of homogeneity index (HI) to the PTV for full‐arc (gantry angle: 181°–180°) and partial‐arc (gantry angle: 181°–360°) VMAT using PTV‐based and robust optimization for three positions. PTV at the periphery position was more homogeneous for the PTV‐based optimization plan than for the robust optimization plan

Position	Technique	PTV‐based	Robustness
Periphery	Full arc	0.015	0.040
	Partial arc	0.033	0.098
Intermediate	Full arc	0.050	0.043
	Partial arc	0.056	0.055
Center	Full arc	0.050	0.047
	Partial arc	0.079	0.057

**Figure 2:**
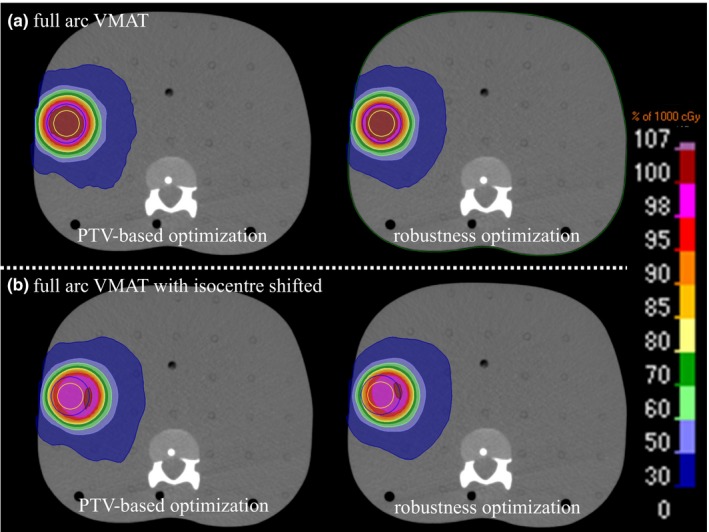
Dose distribution is calculated by the PTV‐based and the robust optimization plans using full‐arc VMAT with the (a) 0 mm and (b) 5 mm isocenters shifted at the periphery position.

**Figure 3:**
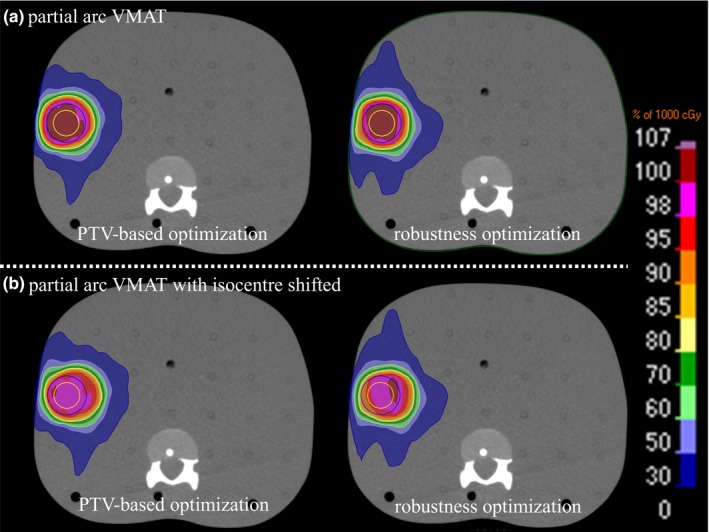
Dose distribution is calculated by the PTV‐based and the robust optimization plans using partial‐arc with the (a) 0 mm and (b) 5 mm isocenters shifted at the periphery position.

**Figure 4:**
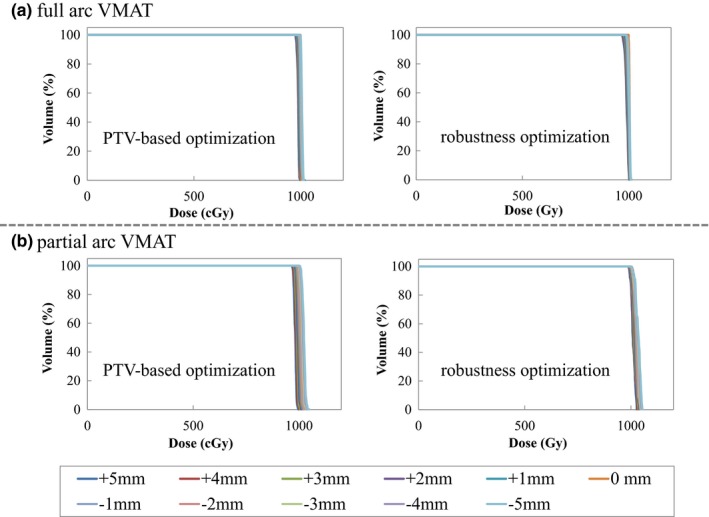
Variation of the DVHs of dose distributions recalculated to the RL directions for PTV‐based and robust optimization plans using (a) full‐arc and (b) partial‐arc VMAT for the periphery position. The robust optimization plan shows less uncertainty than the PTV‐based optimization plan.

Figures 2 and 3 shows the dose distribution for PTV‐based and the robust optimization plans using full‐arc and partial‐arc VMAT at the periphery position. Figure 4 shows the DVH for the effect of the setup uncertainty on the dose distribution for the periphery position in the R‐L direction for PTV‐based and the robust optimization plans using full‐arc and partial‐arc VMAT. The uncertainty in the dose to the CTV using the partial‐arc VMAT plan was dependent on the target's location.

Table 2 compares the D_99%_ doses to the CTV obtained from the rigidly shifted plan between the PTV‐based and the robust optimization plans using partial‐arc VMAT, with data shown as averages and standard deviations, with ranges in parentheses. For the intermediate and center positions, the uncertainties for the D_99%_ doses to the CTV in all directions did not significantly differ when comparing the PTV‐based and robust optimization plans (*P* > 0.05). For the periphery position, uncertainties of the D_99%_ doses to the CTV in the R‐L direction were lower for the robust optimization plans than for the PTV‐based plans (*P* < 0.05). Additionally, uncertainties for the D_99%_ doses to the CTV in the S‐I direction were higher for the robust optimization plans than for the PTV‐based plan (*P* < 0.05).

**Table 2:** D_99%_ doses to the CTV for partial‐ arc VMAT using PTV‐based and robust optimization for three positions. Averages and standard deviations, with ranges in parentheses, are shown

Position	Direction	PTV‐based (cGy)	Robustness (cGy)	*P* value
Periphery	R‐L	999.0 ± 12.2 (979–1016)	999.0 ± 5.4 (991–1006)	0.009^a^
	A‐P	988.5 ± 2.9 (993–1002)	996.8 ± 3.2 (990–1000)	0.364
	S‐I	1000.0 ± 2.4 (995–1003)	985.5 ± 17.2 (950–1003)	0.002^a^
Intermediate	R‐L	994.5 ± 17.2 (956–1011)	992.7 ± 13.6 (964–1006)	0.230
	A‐P	992.8 ± 8.0 (977–1001)	995.8 ± 6.1 (982–1001)	0.213
	S‐I	989.5 ± 2.5 (994–1003)	996.8 ± 4.3 (990–1002)	0.055
Center	R‐L	989.0 ± 10.4 (969–1000)	989.3 ± 11.5 (963–1000)	0.374
	A‐P	988.5 ± 10.1 (971–1000)	992.3 ± 6.9 (981–1001)	0.120
	S‐I	989.5 ± 9.3 (972–1000)	994.7 ± 8.1 (976–1003)	0.331

a The analysis of variance (ANOVA) resulted in a statistically significant variance to the R‐L and S‐I directions in the periphery position (*P* < 0.05).

Table 3 compares the D_99%_ doses to the CTV obtained from the rigidly shifted plan between the PTV‐based and the robust optimization plans using full‐arc VMAT, with data shown as averages and standard deviations, with ranges in parentheses. All positions and directions were no statistically significant differences (*P* > 0.05).

**Table 3:** D_99%_ doses to the CTV for full‐arc VMAT using PTV‐based and robust optimization for three positions. Averages and standard deviations, with ranges in parentheses, are shown

Position	Direction	PTV‐based (cGy)	Robustness (cGy)	*P* value
Periphery	R‐L	990.1 ± 8.2 (977–1000)	987.5 ± 8.3 (972–1000)	0.489
	A‐P	989.0 ± 8.2 (983–1000)	989.3 ± 8.3 (987–1000)	0.471
	S‐I	989.2 ± 8.2 (974–1000)	985.2 ± 8.3 (974–1000)	0.051
Intermediate	R‐L	992.5 ± 7.6 (982–1001)	991.7 ± 6.4 (978–1000)	0.292
	A‐P	989.2 ± 9.8 (972–1000)	986.6 ± 9.7 (969–1000)	0.250
	S‐I	989.8 ± 9.6 (973–1000)	991.4 ± 6.3 (980–1000)	0.095
Center	R‐L	988.6 ± 10.3 (969–1000)	991.1 ± 8.3 (976–1000)	0.498
	A‐P	989.3 ± 9.5 (971–1000)	991.4 ± 7.7 (978–1000)	0.510
	S‐I	988.1 ± 9.9 (971–1000)	989.3 ± 9.2 (974–1000)	0.998

Table 4 compares the HI of CTV obtained from the rigidly shifted plan between the PTV‐based and the robust optimization plans using partial‐arc VMAT, with data shown as averages and standard deviations, with ranges in parentheses. For the periphery position, uncertainties for the HI of CTV in the S‐I direction were higher for the robust optimization plans than for the PTV‐based plan (*P* < 0.05).

**Table 4:** Homogeneity index of CTV for partial‐ arc VMAT using PTV‐based and robust optimization for three positions. Averages and standard deviations, with ranges in parentheses, are shown

Position	Direction	PTV‐based	Robust	*P* value
Periphery	L‐R	0.027 ± 0.002 (0.023–0.030)	0.038 ± 0.003 (0.035–0.045)	0.211
	A‐P	0.027 ± 0.002 (0.025–0.030)	0.042 ± 0.002 (0.040–0.047)	0.269
	S‐I	0.025 ± 0.004 (0.020–0.031)	0.062 ± 0.012 (0.041–0.083)	0.001^a^
Intermediate	L‐R	0.033 ± 0.007 (0.026–0.049)	0.040 ± 0.007 (0.032–0.055)	0.393
	A‐P	0.034 ± 0.006 (0.029–0.047)	0.036 ± 0.005 (0.033–0.048)	0.227
	S‐I	0.034 ± 0.003 (0.030–0.040)	0.035 ± 0.003 (0.031–0.041)	0.475
Center	L‐R	0.019 ± 0.007 (0.012–0.030)	0.039 ± 0.008 (0.024–0.053)	0.266
	A‐P	0.022 ± 0.009 (0.012–0.038)	0.035 ± 0.010 (0.014–0.048)	0.422
	S‐I	0.022 ± 0.009 (0.012–0.041)	0.034 ± 0.008 (0.024–0.050)	0.297

a The analysis of variance (ANOVA) resulted in a statistically significant variance to the R‐L and S‐I directions in the periphery position (*P* < 0.05).

Table 5 compares the HI of CTV obtained from the rigidly shifted plan between the PTV‐based and the robust optimization plans using full‐arc VMAT, with data shown as averages and standard deviations, with ranges in parentheses. All positions and directions were no statistically significant differences (*P* > 0.05).

**Table 5:** Homogeneity index of CTV for full‐arc VMAT using PTV‐based and robust optimization for three positions. Averages and standard deviations, with ranges in parentheses, are shown

Position	Direction	PTV‐based	Robust	*P* value
Periphery	L‐R	0.014 ± 0.004 (0.005–0.017)	0.019 ± 0.006 (0.009–0.030)	0.392
	A‐P	0.017 ± 0.005 (0.005–0.022)	0.017 ± 0.003 (0.009–0.021)	0.226
	S‐I	0.016 ± 0.004 (0.005–0.019)	0.020 ± 0.005 (0.009–0.027)	0.479
Intermediate	L‐R	0.021 ± 0.008 (0.012–0.038)	0.015 ± 0.005 (0.010–0.026)	0.087
	A‐P	0.023 ± 0.009 (0.013–0.039)	0.020 ± 0.009 (0.010–0.036)	0.493
	S‐I	0.022 ± 0.009 (0.013–0.039)	0.016 ± 0.006 (0.010–0.028)	0.106
Center	L‐R	0.021 ± 0.007 (0.013–0.034)	0.018 ± 0.006 (0.011–0.029)	0.286
	A‐P	0.022 ± 0.009 (0.013–0.041)	0.019 ± 0.007 (0.011–0.032)	0.200
	S‐I	0.022 ± 0.009 (0.012–0.038)	0.021 ± 0.009 (0.011–0.036)	0.451

The near maximum dose D_2%_ to the CTV in all positions and directions for the PTV‐based and the robust optimization plans were 1040 and 1068 cGy, respectively.

## 4 DISCUSSION

This study was designed to investigate the possibility of VMAT robust optimization for the homogeneity region. The conventional PTV‐based TPSs for VMAT result in plans that achieve uniform doses to the PTV while minimizing the doses to sensitive structures. In our study, the dose distribution using full‐arc and partial‐arc VMAT within the PTV for periphery locations was more inhomogeneous in the robust optimization plan than in the PTV‐based optimization plan. There was no significant difference in homogeneity between the intermediate‐ and center‐target positions in both full‐arc and partial‐arc VMAT plans. Thus, dose variations for the photon robust plan depend on the location of the tumor as well as the beam's geometry. It should be noted that, in the periphery tumor location, setup error resulted in a difference between the planned and delivered dose distributions. Partial‐arc VMAT has the advantages of decreasing the normal tissue dose, total MU, and delivery time.^2^, ^3^ Partial‐arc VMAT has been widely used for tumors located throughout the body, such as in the lung and the liver, as well as for maxillary cancer treatment to reduce doses in contralateral, healthy organs.

Using partial‐arc VMAT, we applied a setup error in the R‐L direction of the periphery location and found that the standard deviation of the D_99%_ to the CTV was 12.2 cGy and 5.4 cGy for the PTV‐based and the robust optimization plans, respectively, meaning that the uncertainty of the D_99%_ doses to the CTV was lower with the robust optimization plan than with the PTV‐based optimization plan. In contrast, the standard deviation of the D_99%_ to the CTV in the S‐I direction was 2.4 cGy and 17.2 cGy for the PTV‐based and the robust optimization plans, respectively. This phenomenon resulted from compensation for a decreased dose to the CTV in the R‐L direction. It should be noted that tumor motion in the thoracic or upper‐abdominal region results from breathing, which produces the greatest movement in the S‐I direction. Respiratory motion management is one of the important issues in clinical radiotherapy.^17^ The external surface of abdominal wall moves with breathing, and result in a radiological depth variation. Robust optimization is efficacy used in cases where there is likely to be significant radiological path change.^15^ The uncertainty of the D_99%_ doses to the CTV with the robust optimization plan using partial‐arc VMAT was strongly dependent on the target's location. In our study, maximum doses using PTV‐based and robust optimizations were less than 107% of the prescribed dose, were clinically acceptable.

When the setup error occurred in the R‐L direction on the periphery location, the depth of the tumor either increased or decreased. The dose to the target, therefore, would be either lower or higher than the planned dose. When the setup error occurs in the R‐L direction on the periphery location, 360°‐arc VMAT treatment can compensate for the dose to the target from one side even if the dose to the target from the other side is lower. Accordingly, in the periphery location, application of the robust optimization plan resulted in lower uncertainty than that for the PTV‐based optimization plan. Full‐arc VMAT is often used for near center region target. As for the intermediate and center positions, D_99%_ dose and HI to the CTV in all direction using PTV‐based and robust optimization were no statistically significant differences. These results demonstrated that dose to the center region target using Full‐arc VMAT is robust for setup variations.

Because day‐to‐day setup variations were random, it was necessary to use a mathematical calculation of the probability distribution that considers the number of fractions.^18^ The dose distribution within the PTV on the periphery location was more inhomogeneous in the robust optimization plan than in the PTV‐based optimization plan. A more homogeneous plan within the PTV is usually preferred and approved by the physician. If the plan's quality under the robust optimization plan is acceptable, the actual dose to the target can lead to more accuracy. The International Commission on Radiation Units and Measurements (ICRU) noted in Report 24 that the available evidence for certain types of tumors points to the need for 5% accuracy in the delivery of an absorbed dose to a target volume to achieve local control.^19^

The OAR objectives were not constrained in this study. One of the clinical goals depended on the dose to the OAR. The most important goal was that the dose to the tumor was premeditatedly received. An evaluation of the robust optimization plan is thus necessary for preventing large discrepancies between the planned and delivered doses and because robust optimization is a novel method in commercial TPS. Even if PTV‐based optimization is used, we recommend robust evaluation to confirm the uncertainty of doses to the target on the periphery of the body when using partial‐arc VMAT. Plan evaluations should be provided not only for certain‐situation DVHs but also for the several‐situation DVHs to ensure secure therapy.^20^ Another limitation of this study is that it was performed under a phantom study. Further studies are needed to account for nonrigid variations in anatomy and intra‐fractional motion because of the deformations and changes in the positions of anatomical structures daily.

## 5 CONCLUSION

In conclusion, this study demonstrated that the efficacy of the robust optimization plan was dependent on the CTV position. We focused on peripheral cancer, which may benefit from a robust optimization plan due to its location. This study supports the acceptability of robust photon treatment planning based on the dose prescription defined to the CTV.

## CONFLICT OF INTEREST

The authors declare no conflict of interest.
